# A Simple Rule for Proteins to Follow

**DOI:** 10.1371/journal.pbio.1000232

**Published:** 2009-11-03

**Authors:** By Caitlin Sedwick

**Affiliations:** Freelance Science Writer, San Diego, California, Unites States of America

**Figure pbio-1000232-g001:**
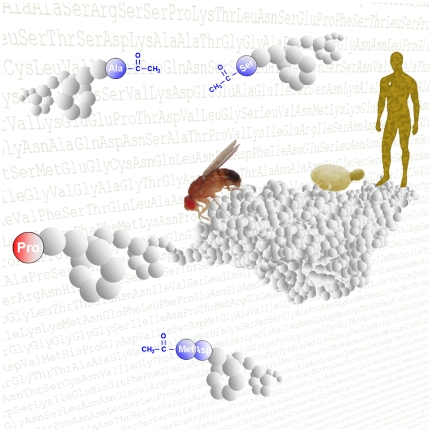
The mechanistic principles of N-terminal protein acetylation are conserved in all organisms investigated so far. With the help of a simple rule to screen for N-terminal acetylation, researchers can now study how this modification affects protein function.

[Fig pbio-1000232-g001]Proteins are the workhorses of cells, acting as enzymes, structural elements, and signal transducers. The tremendous variability in proteins' chemical and physical properties is achieved through the manner in which they are made—by the mixing and matching of a set of basic building blocks known as amino acids. Each amino acid consists of a carbon atom attached to an amine group, a carboxyl group, a hydrogen atom, and one of 22 structurally and chemically distinct side groups. When a cell builds a protein, it uses the instructions encoded in a corresponding gene to tell it which amino acids to use, and in what order. A new protein is assembled front-to-back, with each new amino acid added to the growing chain by hooking its amine group to the carboxyl group of the previous amino acid.

The business of protein assembly is just the first step in creating a functional protein. Once assembled, a protein must next be folded into the proper 3-D shape for its designated cellular function. Then, it may be further altered by a myriad of chemical modifications, which can affect anything from its activity level, to its location within the cell, to its longevity. One such modification, called amino terminal acetylation (N-acetylation), involves adding an acetyl group to the amine group of a protein's first amino acid. N-acetylation affects around 80%–90% of proteins in human cells (and about 50% in yeast). Researchers think N-acetylation occurs as a protein is being made, and that, in some cases, it may affect a protein's proper location, stability, and function within the cell. Until now, the rules governing whether a given protein receives this modification weren't fully understood, a situation rectified in a paper by Sandra Goetze, Erich Brunner, and colleagues in this issue of *PLoS Biology*.

The enzymes that carry out N-acetylation are known as N-terminal acetyltransferases (NATs). They have been identified in yeast and humans and appear to work by recognizing specific sequences of amino acids. However, it turns out that simply being recognized by NATs does not guarantee that N-acetylation will occur; many proteins containing the appropriate recognition sequences lack N-acetylation. Goetze and colleagues postulated that some other feature of proteins overrides NAT recognition to control whether the protein receives N-acetylation.

To explore this possibility, the authors turned to a favorite experimental system—the fruit fly. N-acetylation had been thought to be rare in flies, as few proteins with this modification had so far been identified in invertebrates. But, when Goetze and colleagues looked for N-acetylation on proteins purified from *Drosophila* Kc167 cells, they found that the modification is just as common in flies as it is in yeast and humans.

The researchers searched for patterns in N-acetylation amongst their purified fly proteins. They found that proteins sharing strong sequence similarity within their first 60 amino acids also shared the same N-acetylation state. This prompted them to explore whether the identity and/or order of amino acids within the first two or three amino acids of a protein is the determining factor. Previous work from other groups has shown that certain amino acids in these slots tended to predispose a protein to receive N-acetylation in other eukaryotes. Goetze and colleagues confirmed that this was also true in the fly. But, in addition, they discovered that this predisposition is overruled whenever a particular amino acid—proline—is present in the first or second slot of a protein chain. Proteins can be N-acetylated, it seems, only when there's no proline in either of the front two slots of their amino acid chain. The authors called this simple rule the “(X)PX rule.”

Goetze and colleagues tested the (X)PX rule by making targeted mutations in fly proteins. They took a protein that normally has the inhibitory proline and replaced it with a different amino acid to see whether the protein would then get N-acetylated. They also took a protein that doesn't normally have a proline and swapped one in to test whether it would inhibit N-acetylation. After expressing the modified proteins in cultured fly cells and testing for the presence of N-acetylation by mass spectrometry, they found that both these predictions were borne out. Further experiments using similarly mutated proteins in whole flies also supported the (X)PX rule. Identifying this simple rule now makes it possible to conduct more detailed studies of the role and impact of N-acetylation in cells.

**Goetze S, Qeli E, Mosimann C, Staes A, Gerrits B, et al. (2009) Identification and Functional Characterization of N-Terminally Acetylated Proteins in** ***Drosophila melanogaster*** **. doi:10.1371/journal.pbio.1000236**

